# Solidarity, Afro-communitarianism, and COVID-19 vaccination

**DOI:** 10.7189/jogh.12.03046

**Published:** 2022-08-09

**Authors:** Cornelius Ewuoso, Tom Obengo, Caesar Atuire

**Affiliations:** 1 Steve Biko Centre for Bioethics, University of Witwatersrand, Johannesburg, South Africa; 2 Department of Medicine, University of Cape Town, Cape Town, South Africa; 3 Department of Philosophy and Classics, University of Ghana, Accra, Ghana; 4 Nuffield Department of Medicine, University of Oxford, Oxford, UK

This paper appeals to the Afro-communitarian conceptions of solidarity to think critically about the duty (if any and in what ways) to vaccinate. Barbara Prainsack and Alena Buyx [1] describe solidarity as “enacted commitment to carry the costs (financial, social, emotional, and other contributions) of assisting others with whom a person or persons recognise similarity in a relevant aspect”. In sub-Saharan Africa, one concrete way Africans have enacted this commitment to carry cost is exemplified by the phenomenon of Black tax common in Southern Africa, whereby individuals who are well-off in a family help their indigent relatives.

There are at least two reasons to reflect on the imports of solidarity for the duty to vaccinate. First, some scholars have suggested that COVID-19 vaccine mandates may be justified based on the principle of solidarity [2,3]. Yet, they are hardly clear about the formulation(s) of solidarity (in global or African bioethics literature) that supports this view. This is a critical gap.

Second, global COVID-19 vaccination remains a challenge. More than 50 countries have been unable to (fully) vaccinate at least 10% of their population [4], despite RNA vaccines from Moderna and Pfizer-BioNTech being freely available in most countries, and some studies [4,5] demonstrating their safety and their effectiveness in mitigating severe COVID-19 symptoms and death. This includes (largely populated) African countries like Egypt and Nigeria and low-income countries like Iraq and Afghanistan. Only about 15 (out of 54) African countries have fully vaccinated at least 10% of their population, while only 4.4% of Africans are fully vaccinated [4]. In South Africa, the country with the largest number of COVID-19 cases (about 2.8 million) and deaths (about 87 000) among African countries as of September 27, 2021, the average daily COVID-19 cases remain around 1700 and daily deaths around 200, with most infections and deaths occurring in urban and largely populated areas in Gauteng, Western Cape, and Kwazulu Natal regions [6,7]. Yet, only about 14% of the population has been fully vaccinated. These statistics on vaccination in Africa should be interpreted considering the age structure of the population, but this is outside of the scope of this paper.

Evidently, some factors are responsible for the low vaccination in African countries, including a shortage of doses. Nonetheless, the reluctance to vaccinate has been observed even where doses are available [8]. In fact, vaccine hesitancy is one of the top ten global health threats [9]. Vaccination hesitancy threatens everyone and undermines the global COVID-19 pandemic response and quest to limit and stop the spread of the virus. The global community should not spare any tool for overcoming hesitancy. Any longstanding tradition or culture could offer lessons on the human condition. Africans have cooperated for a long time and formed solidarity with each other. If the principle of solidarity grounded in the African way of being or experiencing the world can help to scale up COVID-19 vaccination, this ought to be explored since *no one is safe until everyone is safe*.

**Figure:**
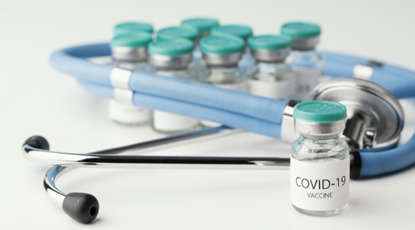
Photo: Corona vaccines. Source: Ali Raza, via https://pxhere.com/en/photo/1640977. Copyright-free under Creative Commons CCO.

In this paper, we argue that the principle of solidarity grounded in Afro-communitarianism implies we ought to vaccinate to further foster vaccine uptake and prevent COVID-19 vaccine-preventable complications and deaths. To justify this thesis, in the first section: a) we highlight some aspects of this principle in African philosophy– namely altruism, reciprocity, and collective responsibility, and b) outline their moral implications for the duty to take safe and effective vaccines. Though we focus mainly on the duty to vaccinate, we do not suggest that vaccination is the *only* means of preventing complications associated with COVID-19. Our analysis equally applies to the duty to socially distance, wear facemasks, or adhere to COVID-19 mitigation protocols. In the second section, we address the objections to our stances on vaccine mandates. Mandates are critical for upscaling vaccine intake, lifting restrictive measures, and limiting complications associated with COVID-19 [2,10]. We demonstrate that, despite attractive aspects of solidarity in African writings favouring voluntary (rather than mandatory) vaccination, it has considerable implications for upscaling COVID-19 vaccination. Following this, we address the objection that the norms we describe do not address the real reasons for vaccination hesitancy. Understanding and addressing these reasons is critical for increasing COVID-19 vaccination. We contend that, though these reasons include a complex combination of confidence, complacency, and convenience, confidence or mistrust in the vaccines appears to be closely associated with reluctance to vaccinate. We then showed how the African view of solidarity implies that scientists should use scientific data and social media to build trust and in what ways they can do so. Finally, we address the objection that the African view of solidarity has no practical relevance for public health ethics or decisions since the motivation for forming solidary groups appears to be essentially self-interested. We demonstrate how this is not problematic since positive reciprocity (solidarity) rather than negative reciprocity (solidarity) tends to be encouraged in Afro-communitarianism.

This paper is different from other studies which focus on understanding the factors affecting COVID-19 vaccine uptake. It is equally different from other studies that appeal to western approaches like consequentialism (or western approaches to solidarity) to reflect on vaccination. Contrarily, the moral implications for the responsibility to vaccinate grounded in solidarity match moral intuitions dominant in the Global South. The idea of solidarity is not unique to African thought and is also found in the west and east. However, the ways of thinking about this concept in African philosophy are based on African modes of being or encountering the world, something Africans did not learn from others.

## AFRICAN DISCUSSIONS ON SOLIDARITY

In African writings, altruistic service or altruism, reciprocity, and collective responsibility to promote the flourishing of all members are common themes often associated with this concept. These themes do not exhaust the concept itself, and a scoping/systematic review is required to map the discussion on solidarity in Afro-communitarianism adequately.

### Altruistic service

The idea that the normative ideal way of exhibiting solidarity entails (an attitude of) altruistic service has been defended by Thaddeus Metz. In Metz's [11] opinion, to exhibit solidarity entails a commitment to act to make others better people, sharing in their failures and successes, advancing their self-realisation for their sake, and improving their situation, having been deeply aware of (and moved by) the other's condition. The one who exhibits solidarity is necessarily sympathetic (s/he feels how others are likely to be impacted having received – or not – our help), empathetic (s/he recognises others who need help and how they require it), and sensitive (s/he does not become indifferent to their needs but thoughtfully respond to them). To fail to exhibit solidarity is either to be indifferent, exhibit ill will, or act in ways that undermine the well-being or the good of others [11]. The core of thinking about solidarity in this way is *responsiveness to others for their sake*. A likelihood of success and acting to benefit others (and not actually succeeding in meeting their needs) are crucial to exhibiting solidarity in this way.

One relevant norm that emerges from this description is that one should act to advance others' good for their sake. The tremendous support and goodwill given to the University of Cape Town (specifically from external donors and agencies, as well as from students and staff who created time in the middle of a tedious semester to participate in the various mop-up operations) following the fire incident that left many students stranded and destroyed some of the university structures like the priceless collection of African studies in the Jagger Reading Room is one concrete way this norm was lived out [12].

What does altruistic service imply for the duty to vaccinate? The pandemic significantly impacted older adults and people of colour. For example, about 50% of older adults living in the rural communities in the United States are believed to be at a higher risk of COVID-19-related complications and death than urban residents, owing to underlying medical conditions, fewer health professionals, less well-resourced health care facilities and a general lack of intensive care and ventilators [13]. Equally, the COVID-19 pandemic has further deepened social inequalities and provoked troubling economic crises for societies and individuals. In South Africa, the pandemic had an enormous impact on many individuals who depend on their daily income. The lockdown measures imposed by the government to curtail the spread of the virus resulted in income loss for many, increasing the number of people on the poverty line [14]. The World Bank predicted that the situation may worsen, with a real possibility that more South Africans (about 45%) will be pushed below the poverty line [14]. There are many ways (such as increasing taxes for the wealthy and allocating helpful economic packages to the poor) to address this problem. Within this context, this way of thinking about solidarity implies we should be responsive to those negatively impacted by the pandemic by vaccinating. This will be a way of demonstrating goodwill to those who have been significantly affected and overturning the gross social inequalities created by this pandemic. With many people vaccinated, governments around the world will be able to lift lockdown measures (as many have done) and begin an economic recovery to allow more people to find jobs and earn an income.

### Reciprocity

Though altruism is one way in which solidarity has been conceptualised in the writings of some African scholars, it does not exclude reciprocal obligations, whereby individuals in a solidary group are *responsive to each other*. A relevant African maxim here is *the right arm washes the left arm and the left arm washes the right arm.* Consider the following remark by Julius Nyerere [15]: “in our traditional African society, we were individuals within a community. We took care of the community, and the community took care of us. We neither needed nor wished to exploit our fellow men.” Similarly, the Shona phrases *kukura kurerwa* and *Chirere chichazo kurerawo* imply that since everyone develops through the contributions of others, everyone should also play a role in the growth of others [16]. The Shona people believe that a community that exhibits solidarity has no orphans, no stepsisters, stepbrothers, or stepmothers. It is one in which we are simply sisters or brothers to one another. The duty this gives rise to is that one’s actions should benefit others because one has been a beneficiary of (or will benefit from) the other’s actions. This duty excludes circumstances where one lacks the means to reciprocate, or where reciprocating reasonably undermines one’s existence or threatens one’s being.

This way of thinking about reciprocity is more positive, whereby one party in reciprocal relations mostly aims to benefit more than others. Precisely, one way of thinking about solidarity in African philosophy is positive reciprocity, such as can be found in the black students' response in 1976 (Soweto uprising) to the introduction of Afrikaans as the medium of learning in black schools. Despite threats of arrests and even deaths, black students who never knew each other began to group based on their racial identity to counter/defeat racist intentions. Mabogo More [17] writes about the grouping of black students in the following way: “This mutual and common comprehension, recognition and appreciation of each other's destinies and projects by black students constitutes reciprocal relations. In such relations, the other becomes an instrument, not for the negation of the self but for its affirmation, the consequence of which is the emergence of group solidarity.” The implication for COVID-19 vaccination is that, as others contribute towards limiting or mitigating the impact and spread of (as well as fatalities associated with) COVID-19 through vaccination, social distancing, wearing facemasks, and adhering to COVID-19 mitigation protocols, we ought to support them by doing the same. The COVID-19 pandemic response will be robust and more effective if we complement each other’s efforts to limit or reduce the virus' spread.

### Collective responsibility

In African philosophical writings, solidarity also tends to entail cooperation among members in ways that imply collective ownership of and responsibility for their (or the community's) destiny, requiring one to contribute her/his best effort to realise the group's aspirations and goals. The Nigerian philosopher Segun Gbadegesin [18] similarly remarks: “every member is expected to consider themselves an integral part of the whole and to play an appropriate role towards achieving the good of all”. While writing about the apartheid atrocities, the former chair of the Truth and Reconciliation commission Archbishop Desmond Tutu [19] also remarks that “*Ubuntu* means that in a real sense even the supporters of apartheid were victims of the vicious system which they implemented (…) The humanity of the perpetrator of apartheid's atrocities was caught up (…) in that of his victim whether he liked it or not”.

This way of thinking about solidarity is rooted in the belief that human lives are deeply interconnected and intricately bound up with one another. As Luis Mbigi [20] retorts: “I cannot separate my humanity from the humanity of those around me”. For Barbara Nussbaum [21], “we are so connected that if you did not sleep well, or if you are not having a good day, how can I sleep well or have a good day?”. This view sharply contrasts Amartya Sen's [22] capability approach, according to which justice or the individual's freedom essentially entails *independence* from others, that is, an individual should value the capability to realise his own functionality regardless of the situation of others. This African view of solidarity implies that one can only discover his identity/freedom through others. The choices and decisions we make impact us all because human reality is interwoven. One’s own identity is connected both to who others say one is and who all of us are together. This is what Dion Forster [23] calls generous ontology: *One's shared identity with others or interconnection with them is itself a condition for the possibility of growth.* One is human to the extent that s/he can be part of a *communal relationship* and others can relate with oneself. For this reason, Lekan Balogun [24] remarks: “solidarity involves commitment, and work, as well as the recognition that even if we do not have the same feeling or the same lives, or the same bodies, we do live [and grow] on common grounds”-

In West Africa, the African motif of the Siamese Crocodile is commonly used to illustrate this. The art depicts two crocodile heads who share a single stomach. Everything one head eats impacts the others [25]. Among the Igbo people of West Africa, one could be held responsible for the debts owed by others (their crimes, failures, and successes). This way, *Ngozi* (an avenging spirit in the Igbo cosmology) punishes the perpetrator of a crime and their relatives, normatively implying that we are accountable for each other [16]. In this way, solidarity is grounded in similarity or solidarity among people; we recognise our shared identity (and not the difference) with the people to whom we exhibit solidarity, our participation in the creation of the problem undermining their dignity, and how our failure to aid them also affect us.

COVID-19 pandemic has revealed that we are not isolated humans. When we undermine the well-being of others, we undermine our well-being because we are implicated in one another's lives. This view gives rise to a variety of norms. But relevant here is the idea that we have a responsibility and a debt to promote the flourishing of each other – within this context – by vaccinating since those who do not vaccinate (or observe other measures for mitigating the spread of COVID-19) bar any underlying medical conditions that may prevent them from vaccinating, put our lives and their own lives, as well as our collective capacity to recover from the COVID-19 pandemic, at risk.

## MANDATORY VACCINATION AND SOLIDARITY

In this section, we address some objections against our position. A vaccination enthusiast will be correct to point out that the conceptions of solidarity described above do not imply that COVID-19 vaccination should be mandated to increase its uptake or limit the real impact of the virus. This appears to be the view of Julian Savulescu [2] and Keymanthri Moodley [3], whose primarily consequentialist justification for compulsory vaccination includes a grave threat to public health by the unvaccinated, lower vaccine risk or high confidence in vaccine safety/effectiveness, and greater expected utility of vaccination in comparison to the alternatives. The fatality rates in some countries (2530 in the United States, 676 in Brazil, 846 in Russia) remain high as of September 30, 2021. Other countries like Tanzania and Botswana have been able to limit the real impact the virus could have and bring the daily death and infection rates under control through legally enforced restrictive measures and lockdowns [26]. However, the catastrophes (economic, health systems, and others) resulting from these restrictive measures have been enormous. Eventually, governments would have to ease restrictions, an action with its own implications. The global community would witness a resurgence of fatalities due to COVID-19. For example, with the government of South Africa easing the lockdown measures, experts think a 5th wave might occur soon in the country. Vaccination could help prevent reinfections. Globally, vaccination is recognised as the *most effective* public health measure for limiting the spread of vaccine-preventable infections, saving lives (or preventing harm). Failure to increase vaccine uptake could potentially undermine the well-being of vaccinated individuals, since immunity wanes over time. Contrarily, compelling vaccination (through in/direct consequences) appears to be a reasonable way (and better alternative) of *quickly upscaling* vaccination and *reducing* the burden to health care systems (a major reason for restricting liberties). Others may critique that we have merely focused on the attractive formulations of solidarity in Afro-communitarianism that supports voluntary vaccination. There may be other accounts of solidarity that could justify vaccine mandates.

In response, we acknowledge that if many people are immune, the capacity of the virus to spread is significantly reduced. Evidently, empirical data from Canada, France and the United States support an increased vaccine uptake trend in populations to whom vaccine mandates apply [27]. But empirical uncertainty remains on whether vaccine mandates in Africa will lead to vaccine uptake on the continent. If social values and preferences are strong influencers of health behaviours [28], then one should tread cautiously when appealing to empirical data outside of Africa to inform decisions and policies in Africa, and for Africa, since as scholars have shown Africans tend to live by a different value system [29]. Accordingly, understanding people's social values and preferences can become a powerful tool for formulating policies regarding vaccination, increasing vaccine confidence and upscaling COVID-19 vaccinations. Health campaigns for COVID-19 vaccination uptake will be more effective if they take seriously how health behaviours are influenced. In fact, empirical data obtained from the continent reveals an existing increase in positive attitudes to COVID-19 vaccines in African countries like South Africa [30]. The positive disposition suggests that vaccine mandates may be unnecessary for upscaling vaccine intake, at least in these countries. Additionally, this disposition towards COVID-19 vaccines appears to also demonstrate that most Africans value solidarity. They seem to recognise the imperative to act altruistically to improve the conditions of those most vulnerable to the virus. They are willing to take steps to limit the exposure of others to the virus because they see themselves implicated in their lives, and how the failure to act (or to vaccinate) also affects them.

Regarding whether we have merely focused on the aspects of solidarity that appeal to us, we acknowledge that the formulations of solidarity and its implications we used do not *perfectly* represent *all* the ways solidarity is conceptualized in African writings. Though we have drawn selectively on some formulations of this concept, we do not think this is problematic since our goal is to appeal to its more salient formulations in African philosophy to describe how African scholars are more likely to think about the duty to vaccinate. Also, many African scholars believe that cooperation is partly valuable when people willingly come together to share a way of life, stay together, and seek others' well-being for their sake [31]. This has practical relevance for upscaling vaccination. A sense of shared identity has been found to be essential to, as well as positively impact, how individuals cooperate to respond to threats like the COVID-19 pandemic [32]. Additionally, it seems intuitive that altruism is hardly *altruism* if it is compelled. Solidarity is also *valuable partly when* individuals in the solidary group can believe that others will not disappoint them or take advantage of their “naivety”. Trust enables individuals in the solidary group to share a way of life or identity and cooperate to realise the shared vision, even when success may be uncertain. It seems to us that any formulation of solidarity entailing vaccine mandates will likely imply that we should promote solidarity however we can. Such formulation would imply authoritarian approaches that would result in willing sacrifices of individual rights and liberties with the goal of promoting solidarity. Many African scholars believe that this is not the right way to showcase humanity [30]. In other words, we have a duty to promote communal relationships through friendly means rather than maximise or promote it regardless of the consequences. Nonetheless, as we have acknowledged previously, a systemic review is required to map the discussion on solidarity in African philosophy adequately.

## UNDERLYING REASONS FOR VACCINE RELUCTANCE AND SOLIDARITY

Another objection to the norms described in this paper is that they do not address the prevailing reasons for refusing vaccination. These reasons tend to include a complex combination of confidence, complacency, and convenience. But COVID-19 hesitancy is most closely associated with mistrust or lack of confidence in COVID-19 vaccines due to their risks and side effects (more than their effectiveness). The rushing of COVID-19 vaccine development has had a negative impact on the public's confidence in the vaccine [33]. Hesitancy is also associated with mistrust in the government and the health care system. In fact, studies show that the public will not receive the COVID-19 vaccine if they do not trust the health care system, the authority, and the communication response from the government and scientific community, implying that trust is a significant driver of upscaling vaccination. Most of those who have expressed a lack of confidence also identified social media as their main source of information [10,34]. A theory that aims to foster vaccine uptake should address these reasons, particularly trust.

This objection raises essential questions about communication and information dissemination since these will be necessary to reassure the public's confidence in vaccine safety. Communication strategies are required to realise these goals, which we will address this in a future study. The objection equally raises essential questions about how scientists ought to respond to concerns (including conspiracy claims) in a way that is accountable and promotes public trust. Additionally, it raises critical questions about what continues to motivate science, and to what extent these motivations are open to public scrutiny. The idea is that these motivations must be open to scrutiny because they are essential in allowing the public to make right moral judgements about vaccination or trusting science. Put differently, the public will hesitate to cooperate with scientists and governments to upscale vaccination if they do not trust them, whereby trust is understood as the readiness to be vulnerable because one believes others will do no harm. Evidently, social media plays a role in sustaining mistrust and fostering misinformation (through conspiracy or false theories) and inadequate information about COVID-19 vaccines and their effectiveness [10]. However, it can also be a tool for education. For example, 31% of persons who said they would not receive the COVID-19 vaccine trust social media [10]. Scientists and governments should use this medium to disseminate scientific data that counter inaccurate information and build public trust in vaccines. Since no vaccine is 100% effective, some side effects have been reported in individuals who have received COVID-19 vaccination. However, these cases are rare and do not outweigh the expected utility; most COVID-19 deaths or complications continue to occur among the unvaccinated [35]. Trust-building measures should also include honest engagement with claims, including claims that challenge scientists' views.

Using scientific data/facts to counter false information and build trust through social media is not enough. Some individuals are more likely to believe persons they know and trust and with whom they have formed long-standing relationships and co-operations; within the African settings, these persons are elders, traditional leaders, clerics, pastors and imams. The principle of solidarity grounded in Afro-communitarianism suggests that these trusted individuals should participate in trust-building, COVID-19 vaccination uptake measures, and campaigns to make them more effective.

## SOLIDARITY AND PUBLIC HEALTH DECISIONS

Someone could claim that solidarity encourages individuals to consider the long-term impact an action may have on them or evaluate their decisions through the lens of egoistic calculus. For this reason, it seems not to be a good principle for public health ethics. When activists and ethicists appeal to individuals to take COVID-19 vaccines because solidarity requires this, it is not merely for “altruistic” reasons or because they genuinely care for the well-being of others. Instead, it is because this costs less than the later intensive care that may be required due to COVID-19 complications or the burden on the health system. This can be compared to the Prisoner's Dilemma thought experiment [36]. Whether it is the revised version of the experiment, where there is a possibility for retaliating non-cooperation or where only one turn exists and decisions are made anonymously, if parties can communicate with one another, however briefly, rationally self-interested individuals will cooperate. The point here is that social solidarity appears to be engendered by self-interests. “If solidarity can form, it will, and the logic of self-interest will be submerged as a result” [37]. This appears not to be a good principle for public health decisions, which may require acting for the common good than our self-interests.

It seems intuitive that self-interested theories like egoism or subjectivism can be a basis for public health decisions in the same way other-regarding approaches like relationalism could be used to realise one's self-interests. Within this context, this implies that one ought to distinguish between how Afro-communitarians have understood this principle and how others may employ it. It seems intuitive that these two are different, and in fact, bad people may use good principles to serve their interests. This would not be a problem with the principle itself, but how individuals have employed it and to what end. While we acknowledge that the thinking about solidarity as entailing reciprocal obligation entails elements of self-interests, altruism as the basis for solidarity (as previously discussed) is not equally unusual in African philosophy. This pro-social behaviour occurs frequently enough, implying that the thinking about solidarity in African writings can adequately motivate public health decisions. The reader should equally note the distinction we made between negative reciprocity and positive reciprocity, wherein positive reciprocity that requires mutual aid is what is encouraged. Precisely, positive reciprocity implies that the basis for forming/joining solidary groups is *not to gain/get more* than others. Rather, reciprocity entails an exchange of actions, whereby an act that *positively impacts* others is reciprocated with an approximately equal positive act.

Finally, the reader should note that we did not aim to determine which formulation of solidarity in African philosophy is *the best or the most plausible*. Instead, we have focused on the *implications of dominant* formulations of solidarity in African philosophy for the duty to vaccinate. Notwithstanding, many Africans tend to think that reciprocity is a strong motivation for action and a valuable public health ethics principle, as evidenced in some forms of relationships like *letsema*, whereby members in a solidary group assist one another to harvest crops and equitably share the produce. Participation in this relationship is grounded in the thinking that prescribes reciprocal relationship (or entails an expectation of reciprocity). Those who assist others in harvesting their produce appear to do so, hoping that others will do the same or support them at the time of their harvest. Additionally, one study [38] that explores the preferences of adolescents, parents, and caregivers participating in an HIV-TB genomic study in Botswana regarding genetic findings indicates that the majority of these Africans consider the obligation to reciprocate participation in genomic research by returning actionable findings, an ethical necessity.

## CONCLUSION

In this paper, we have argued that the principle of solidarity in Afro-communitarianism tend to favour voluntary, rather than mandatory, COVID-19 vaccination. Thus, if vaccination can be mandated from the Afro-communitarian perspective, solidarity appears to be an inadequate principle to justify this position. In a previous publication [39], one of us demonstrated how ethics of friendliness grounded in a modal relational African account may justify the use of coercion or unfriendliness. In this regard, mandatory vaccination may be justified if it is necessary for the operational requirement of a workplace or accessing public facilities, and there is no other way to ensure public safety or prevent harm. Contrarily, if the African views of solidarity were to be the default consciousness of humanity, the implications for the duty to vaccinate are that individuals will voluntarily vaccinate, 1) because we ought to act for others (disproportionately affected and vulnerable individuals) sake, 2) within the context of the pandemic, this will be an appropriate way to act for the well-being of others and expect others to act for one's well-being, and 3) we are implicated in one another's lives and conditions such that the failure to act for their good, entails a failure to be solidaristic. Studies are still required to inquire about how these norms can form the basis of public health ethics or decisions during a pandemic.
